# Protein truncating variants in *FANCM* and risk for ER-negative/triple negative breast cancer

**DOI:** 10.1038/s41523-021-00338-1

**Published:** 2021-09-28

**Authors:** Paolo Peterlongo, Gisella Figlioli, Andrew J. Deans, Fergus J. Couch

**Affiliations:** 1 Genome Diagnostics Program, IFOM – The FIRC Institute for Molecular Oncology, Milan, Italy; 2 Genome Stability Unit, St Vincent’s Institute, Fitzroy, Victoria, Australia; 3 Departments of Health Sciences Research, Laboratory Medicine and Pathology, and Oncology, Mayo Clinic, Rochester, USA

**Keywords:** Genetic association study, Breast cancer, Cancer genetics

## Abstract

*FANCM* protein truncating variants (PTVs) are emerging as risk factors for ER-negative and triple negative breast cancer. Here, we discuss evidence that greatest risk associates with PTVs, such as p.Arg658*, that extensively truncate the 2048 amino acid FANCM protein. Moreover, risks associated with other less-truncating FANCM PTVs such as p.Gln1701* and p.Gly1906Alafs12* may be amplified by additional gene variants acting as modifiers. Further studies need to be conducted taking into considerations these aspects.

Germline variants in the *BRCA1* and *BRCA2* genes are associated with a high-risk of breast and ovarian cancers. These genes are now widely screened to assess the risk of developing these diseases in variant carriers and their relatives. Over the last 15 years, protein-truncating variants (PTVs) in several other genes have been described or proposed to cause moderate- to high-risk for breast cancer but insufficient data hamper a conclusive annotation. Early this year, two large case-control studies were published in the New England Journal of Medicine, which aimed to clarify which genes should be used clinically and to estimates the breast cancer risk magnitude associated with PTVs in each gene. These studies were based on the sequencing of 34 and 28 known or candidate breast cancer risk genes in 113,000 women from 25 countries, and 64,000 women from the US, respectively^1,2^. Both studies found that PTVs in the 8 genes *BRCA1, BRCA2, PALB2, BARD1, RAD51C, RAD51D, ATM*, and *CHEK2* were significantly associated with breast cancer risk. In particular, PTVs of *ATM* and *CHEK2* were associated with risk of estrogen receptor (ER) positive breast cancer, and PTVs of *BARD1, RAD51C,* and *RAD51D* with a risk of ER-negative breast cancer. Moreover, among the genes previously proposed to be associated with an increased risk of breast cancer, the majority including *NBN* and *RECQL*, were found not associated. The unparalleled statistical power of such a large dataset appears to make this a definitive designation^3,4^. But for one of the candidate genes, *FANCM*, the role in breast cancer predisposition appears more nuanced. In the Dorling et al. study, *FANCM* PTVs were not found to be associated with overall breast cancer risk nor to have an effect on age at diagnosis, but were statistically associated with risk in ER-negative breast cancer cases (Table 1). In the Hu et al. study, no association was found in the overall analysis between *FANCM* pathogenic variants and breast cancer risk, and in other association analyses (Table 1). However, a sensitivity analysis for the influence of each study on odds ratios (ORs) for associations between PVs and breast cancer risk detected non-significant odds ratios consistently higher than 1 suggesting that larger studies would be required to reach statistical association, if such exists.

**Table 1:** Results from association studies conducted in European cases and controls on *FANCM* PTVs and breast cancer risk ordered as mentioned in the text.

Reference	Study cohort	Seq./PTV genotyping	Group	Carriers/Non-carriers (freq%)	OR (95% CIs); P-value
Dorling et al.^1^	Pop-based,Europeans	Gene Seq.	Controls	300/50,403 (0.60)	-
			All cases	302/48,524 (0.62)	1.06 (0.90–1.26); 0.48
			**ER-neg cases**	**57/7709 (0.73)**	**1.39 (1.04–1.86); 0.028**
			**TNBC cases**	**23/2841 (0.80)**	**1.64 (1.07–2.53); 0.025**
Hu et al.^2^	Pop-based,Europeans (75%)	Gene Seq.	Controls	46/32,498 (0.14)	-
			All cases	51/32,196 (0.16)	1.14 (0.76–1.74); 0.52
			ER-neg cases	10/3795 (0.26)	1.57 (0.70–3.22); 0.24
			TNBC cases	5/1458 (0.34)	1.71 (0.50–4.49); 0.33
Gracia-Aznarez et al.^5^	Fam-based, Europeans	c.5791 C >T, p.Gly1906Alafs12*	Controls	5/3891 (0.13)	-
			All cases	10/3399 (0.29)	2.29 (0.71–5.54); 0.13
Peterlongo et al.^6^	Fam-based, Europeans	c.5791 C >T, p.Gly1906Alafs12*	**Controls**	**4/6621 (0.06)**	**-**
			**Fam cases**	**18/8617 (0.21)**	**3.93 (1.28–12.11); 0.017**
Kiiski et al.^7^	Pop-based (69%),Fam-based (31%), Finnish	c.5791 C >T, p.Gly1906Alafs12*	Controls	8/2726 (0.29)	-
			All cases	28/4778 (0.58)	1.94 (0.87–4.32); 0.11
			Fam cases	8/1223 (0.65)	2.50 (0.83–7.51); 0.10
			ER-neg cases	5/733 (0.68)	2.34 (0.75–7.35); 0.14
			**TNBC cases**	**5/320 (1.54)**	**5.14 (1.65–16.0); 0.005**
Kiiski et al.^8^	Pop-based (68%),Fam-based (32%), Finnish	c.5101 C >T, p.Gln1701*	Controls	38/2042 (1.83)	-
			**All cases**	**96/2983 (3.12)**	**1.86 (1.26–2.75); 0.0018**
			**Fam cases**	**45/1282 (3.39)**	**2.11 (1.34–3.32); 0.0012**
			**ER-neg cases**	**21/526 (3.84)**	**2.37 (1.37–4.12); 0.0021**
			**TNBC cases**	**12/192 (5.88)**	**3.56 (1.81–6.98); 0.0002**
Neidhardt et al.^9^	Fam-based,Germans	Gene Seq.	Controls	11/2176 (0.50)	-
			Fam cases	21/2026 (1.03)	2.05 (0.94–4.54); 0.049
			**EO fam cases**	**17/1376 (1.22)**	**2.44 (1.08–5.59); 0.02**
			**Fam TNBC**	**4/211 (1.86)**	**3.75 (1.00–12.85); 0.02**
Girard et al.^10^	Fam-based, French	Gene Seq.	Controls	3/1196 (0.25)	-
			Fam cases	7/1200 (0.58)	2.3 (0.6–9.0); 0.23
Figlioli et al.^11^	Pop-based (86%),Fam-based^ (14%), Europeans	c.1972C >T, p.Arg658*	Controls	19/53,717 (0.03)	-
			All cases	31/67,038 (0.05)	1.26 (0.71–2.25); 0.43
			**ER-neg cases**	**10/10,750 (0.09)**	**2.44 (1.12–5.34); 0.034**
			**TNBC cases**	**7/4794 (0.15)**	**3.79 (1.56–9.18); 0.009**
		c.5101 C >T, p.Gln1701*	Controls	122/53,635 (0.23)	-
			All cases	155/66,951 (0.23)	1.09 (0.85–1.38); 0.798
			ER-neg cases	21/10,748 (0.19)	0.97 (0.61–1.56); 0.369
			TNBC cases	10/4794 (0.21)	1.09 (0.57–2.10); 0.149
		c.5791 C >T, p.Gly1906Alafs12*	Controls	96/53,633 (0.18)	-
			All cases	116/66,968 (0.17)	1.05 (0.80–1.38); 0.731
			ER-neg cases	27/10,742 (0.25)	1.52 (0.98–2.35); 0.070
			TNBC cases	10/4795 (0.21)	1.29 (0.67–2.50); 0.461

Abbreviations: *Seq* sequencing, *PTV* protein-truncating variant, *freq* carrier frequency, *OR* odds ratio, *CI* confidence interval, *Pop* population, *Fam* familial cases tested negative for *BRCA1/2* pathogenic variants, *ER-neg* estrogen receptor-negative, *TNBC* triple-negative breast cancer, *EO* early-onset (<51 years).

^ Great majority of these familial cases are untested for *BRCA1/2* pathogenic variants. Statistically significant results are in bold.

Similarly to the findings of Dorling et al. and Hu et al. studies, data from previously published association studies conducted on European cases and controls generally indicated a lack of association between *FANCM* PTVs and breast cancer risk but found associations with ER-negative or triple-negative breast cancer (TNBC) disease subtypes (Table 1). Historically, *FANCM* was implicated in breast cancer risk in 2013, based on exome sequencing of six multi-case breast cancer families and the resulting identification of the *FANCM*:c.5791 C >T PTV in an Italian proband. The variant genotyping in breast cancer cases and controls revealed, however, a non-significant OR^5^. Subsequently, c.5791 C >T was found to be significantly associated with breast cancer risk in European familial cases and in Finnish TNBC cases^6,7^. The *FANCM*:c.5791 C >T variant is unusual in that it does not cause the Arg1931STOP amino acid (aa) change expected according to the genetic code. Instead, as we showed in cell-based experiments, the variant creates a consensus sequence for a splicing silencer triggering the skipping of exon 22. This causes a frameshift starting at aa position 1906 forming a stop codon 12 codons later^6^. Therefore, while the c.5791 C >T variant is universally known as p.Arg1931*, the correct protein annotation of this variant would be p.Gly1906Alafs12*. *FANCM*:c.5101 C >T (p.Gln1701*) is another PTV identified in a Finnish population study, by exome sequencing of 11 breast cancer families. Further genotyping of this allele found it to be significantly associated with breast cancer risk with higher ORs detected in ER-negative and TNBC cases^8^. In addition to single PTVs genotyping studies, two burden analyses of all *FANCM* PTVs discovered by gene sequencing were published^9,10^. Altogether, these data indicate that, while the association with risk for breast cancer was inconclusive, *FANCM* PTVs might be risk factors for familial breast cancer or for ER-negative and TNBC disease subtypes (Table 1). However, these inconclusive results and different risk estimates could be due to study design considerations such as population stratification and ascertainment biases.

More recently, we tested *FANCM*:p.Gln1701*, p.Gly1906Alafs12*, and c.1972C >T (p.Arg658*), which is the third most common *FANCM* PTV found in Europeans, in a large study including breast cancer cases and controls from 19 European countries. Surprisingly, we only found p.Arg658* to be associated with risk of ER-negative and TNBC subtypes^11^. We also functionally tested these three PTVs for their capacity to rescue survival and chromosome fragility in a *FANCM*^*−/−*^ patient-derived cell line exposed to the DNA damaging agent diepoxy butane. We observed that p.Arg658* was unable to rescue the cell survival and chromosome fragility while p.Gln1701* and p.Gly1906Alafs12* showed an intermediate effect^11^. This is consistent with the fact that the p.Gln1701* and p.Gly1906Alafs12*, located in the C-terminus of the protein, are both expected to just disrupt the binding domain of the partner protein FAAP24; while p.Arg658*, located much upstream in the gene, is expected to cause the loss of greater protein portions. Based on these genetic and functional data, it is possible to speculate that the effects on breast cancer risk of the different *FANCM* PTVs might be due to the extent of protein truncation of the PTV. In support of this hypothesis, two women with early-onset breast cancer (at age 29 and 32); were genotyped as homozygotes for p.Arg658*. In addition, one developed several cancers, and the other demonstrated chromosomal fragility^12^. In the same study, two women genotyped as homozygotes for p.Gln1701*, and one homozygous for p.Gly1906Alafs12*, developed breast cancer at age 52 or later, and their cells did not demonstrate chromosome fragility.

As FANCM protein is a large, multi-domain anchor that binds both DNA and other protein complexes at DNA damage sites, different effects may arise relating to breast cancer predisposition, depending upon the protein domains deleted. For example, p.Gln1701* and p.Gly1906Alafs12* delete the C-terminal DNA binding domain and FAAP24 interaction site but retain interaction with two complexes necessary for DNA repair (the Fanconi Anemia core complex and the Bloom syndrome complex). These protein complexes associate at distinct regions located between aa 960–1210^13^ which are lost in the Arg658* variant protein. Previous in vitro work has shown that the ability to recruit Fanconi and Bloom syndrome complexes is more important to the function of FANCM protein than the association with FAAP24^13^. Furthermore, although yet to be experimentally validated, Arg658* can be predicted to act in a dominant-negative fashion. Because the Arg658* protein variant retains the N-terminal DNA binding and ATPase domain, it could potentially block the recruitment of DNA repair complexes by the full-length protein in heterozygotes and reduce DNA repair capacity. In most other breast cancer genes, including *BRCA1* and *BRCA2*, it is the loss of DNA repair capacity that associates with variant severity.

For *BRCA1*, *BRCA2,* and other genes associated with breast cancer, there is also an impact of population ancestry and potential modifier alleles. Could this explain the differences between studies investigating *FANCM* variants and their impact on breast cancer? First, the Finnish studies were based on the p.Gln1701* and p.Gly1906Alafs12* genotyping in geographically, ethnically, and genetically matched cases and controls. In addition, the p.Gln1701* and p.Gly1906Alafs12* carrier frequencies are high in Finns being 1.62% and 0.92%, vs. 0.21% and 0.21% in non-Finnish Europeans (https://gnomad.broadinstitute.org/), respectively. This has allowed the authors of these studies to derive robust associations with ER-negative and TNBC risk in a relatively small sample size. Second, the multicentric study was based on greater case-control numbers allowing to reach a statistical power sufficiently large to show that p.Arg658*, despite a low carrier frequency of 0.033% in non-Finnish Europeans (https://gnomad.broadinstitute.org/), was associated with ER-negative and TNBC risks^11^. It is hence puzzling that in this study no association was found for p.Gln1701* and for p.Gly1906Alafs12* and risk for breast cancer or the ER-negative and TNBC disease subtypes. One potential explanation is the relative genetic isolation of the Finnish population. Finns have recurrent *BRCA1/2* founder PVs and at least 12 other PVs in the moderate risk genes *ATM, CHEK2, FANCM, PALB2, RAD51C,* and *RAD51D* have known that account for the majority of Finnish breast cancer cases with a genetic component^14^. In addition, an excess of double heterozygosity for variants in the moderate penetrance genes has also been documented in Finns^14^. It can therefore be speculated that some other modifier allele exists within this population which amplifies the p.Gln1701* and p.Gly1906Alafs12* risk effects, although the studies to locate such a modifier have yet to be conducted.

In conclusion, all these observations indicate that certain *FANCM* PTVs could be moderate risk factors for ER-negative and TNBC. It is possible that deletions that remove more of the FANCM protein, such as p.Arg658*, are likely to have a stronger effect on both the DNA repair defect and the risk for ER-negative or TNBC. Conversely, the risk effects of *FANCM*:p.Gln1701* and p.Gly1906Alafs12* on these breast cancer subtypes are probably lower but may be amplified by additional variants acting as modifiers. Importantly, the two new very large studies from Dorling et al. and Hu et al. have not analyzed each *FANCM* PTVs singularly nor grouped based on their location within the gene. More clear results about which of the *FANCM* PTVs are moderate risk factors for ER-negative or TNBC and which are not may arise if this were to be done.

## Data Availability

No novel data are shown in this commentary. All data described here have been previously published.
